# Addressing data gaps on long-term care in Canada through data linkage

**DOI:** 10.23889/ijpds.v9i5.2872

**Published:** 2024-09-18

**Authors:** Kristyn Frank

**Affiliations:** 1 Statistics Canada

## Background

Within the context of Canada’s aging population, the complexities of its long-term care system, and the COVID-19 pandemic, information about long-term care facilities, their residents, and their workers is needed. However, little information exists on long-term care residents as they are rarely included in population surveys, and data on the facility characteristics of long-term care workers’ places of employment are often not available.

## Objective and Approach

To effectively monitor changes, improvements, and health outcomes in Canada’s long-term care sector, many data gaps need to be addressed. This paper will present the results of an initiative that sought to examine the potential of integrating existing survey and administrative data sources to fill these gaps. A novel approach was taken which focused on how both facility-level and individual-level data could be integrated, allowing for a more comprehensive understanding of long-term care in Canada.

## Results

This initiative resulted in the development of two data linkage proposals. This presentation will provide an overview of the data sources identified for linkage, the approach taken to examine the feasibility of these linkages, challenges with integrating the data sets, and next steps to move the initiative forward.

## Implications

e data linkages that will be discussed will help to address data gaps on Canada’s long-term care system. The proposed data linkages may also provide researchers with ideas for using similar data sources to address data gaps in long-term care in their respective countries.

